# Characterizing semi-directed phylogenetic networks and their multi-rootable variants

**DOI:** 10.1007/s12064-025-00453-8

**Published:** 2025-12-10

**Authors:** Niels Holtgrefe, Katharina T. Huber, Leo van Iersel, Mark Jones, Vincent Moulton

**Affiliations:** 1 Delft Institute of Applied Mathematics, Delft University of Technology, Delft, The Netherlands; 2 School of Computing Sciences, University of East Anglia, Norwich, United Kingdom

**Keywords:** Mixed graph, Semi-directed phylogenetic network, Tree-based network, Tree-child network, Orchard network, Path partitions

## Abstract

In evolutionary biology, phylogenetic networks are graphs that provide a flexible framework for representing complex evolutionary histories that involve reticulate evolutionary events. Recently, phylogenetic studies have started to focus on a special class of such networks called *semi-directed networks*. These graphs are defined as mixed graphs that can be obtained by de-orienting some of the arcs in some *rooted phylogenetic network*, that is, a directed acyclic graph whose leaves correspond to a collection of species and that has a single source or root vertex. However, this definition of semi-directed networks is implicit in nature since it is not clear when a mixed-graph enjoys this property or not. In this paper, we introduce novel, explicit mathematical characterizations of semi-directed networks, and also *multi-semi-directed networks*, that is mixed graphs that can be obtained from directed phylogenetic networks that may have more than one root. In addition, through extending foundational tools from the theory of rooted networks into the semi-directed setting—such as cherry picking sequences, omnians, and path partitions—we characterize when a (multi-)semi-directed network can be obtained by de-orienting some rooted network that is contained in one of the well-known classes of tree-child, orchard, tree-based or forest-based networks. These results address structural aspects of (multi-)semi-directed networks and pave the way to improved theoretical and computational analyses of such networks, for example, within the development of algebraic evolutionary models that are based on such networks.

## Introduction

**Fig. 1:**
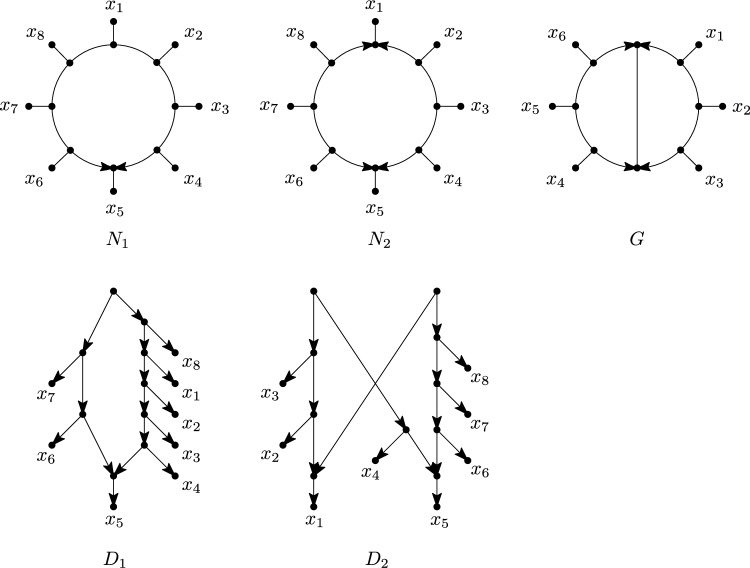
Five mixed graphs $N_1,N_2,G,D_1,D_2$. Mixed graph $N_1$ is a semi-directed network since it is the semi-deorientation of, for example, the rooted network $D_1$ illustrated below it. Mixed graph $N_2$ is a multi-semi-directed network since it is the semi-deorientation of, for example, the multi-rooted network $D_2$ illustrated below it. However, it can be shown that $N_2$ is not a semi-directed network (since two roots are needed). The mixed graph *G* is not semi-directed nor multi-semi-directed

Phylogenetic networks are a generalization of evolutionary trees that are used to represent evolutionary histories of organisms such as plants and viruses that can evolve in a non-tree-like fashion (see e.g. Huson et al. 2010). In particular, they permit the representation of *reticulate* events, in which, for example, two species cross with one another or transfer genes. There are several classes of phylogenetic networks, but in this paper we will mainly focus on rooted phylogenetic networks (see e.g. Kong et al. 2022 for a recent overview), and some of their recent generalizations. Essentially, a rooted phylogenetic network is a directed acyclic graph, usually with a single source or *root*, whose leaf set corresponds to some collection of species or taxa. For example, in Fig. 1, $D_1$ is a rooted phylogenetic network for the collection $\{x_1,\dots ,x_8\}$ of species. Lately, *multi-rooted networks* have also become of interest, which only differ from rooted phylogenetic networks in that they may have multiple roots (see e.g. $D_2$ in Fig. 1). Such networks can be used to model ancestral relationships between populations (Soraggi and Wiuf 2019) and for representing the evolutionary history of distantly related groups of species that can still exchange genes (Huber et al. 2022; Scholz et al. 2019). In both types of networks, vertices with indegree greater than 1 are of special interest because they represent reticulate events. Hence, such vertices are commonly called *reticulations* and their incoming arcs *reticulation arcs*.

Recently, a new class of phylogenetic networks called *semi-directed networks* (Solís-Lemus and Ané 2016) has started to receive a lot of attention in the literature, both from a theoretical (e.g. Baños 2019; Gross and Long 2018; Gross et al. 2021; Linz and Wicke 2023; Jingcheng and Ané 2023; Englander et al. 2025) and applied (e.g. Allman et al. 2025b; Holtgrefe et al. 2025) point of view. Basically speaking, these are mixed graphs (i.e. graphs that can have a combination of undirected edges and directed arcs), which can be obtained by partly deorienting a rooted phylogenetic network, that is, by replacing all arcs which are not reticulation arcs with an edge, and then suppressing any resulting degree-2 vertex that arises from a root. We call such a partial deorientation, in which only reticulation arcs keep their direction and the root locations are lost, a *semi-deorientation*. For example, in Fig. 1, $N_1$ is a semi-directed network since it is the semi-deorientation of, e.g., the rooted network $D_1$. In this paper, we will also consider *multi-semi-directed networks*, that is, mixed graphs which are the semi-deorientation of some multi-rooted network. Note that these networks were recently considered in Maxfield et al. (2025) in the context of defining a dissimilarity measure between semi-directed networks. As an example, in Fig. 1, $N_2$ is multi-semi-directed since it is the semi-deorientation of $D_2$. In general, in case a (multi-)semi-directed network *N* is a semi-deorientation of a (multi-)rooted network *D*, we shall call *D* a *rooting* of *N*.

Locating the root in a phylogenetic network inferred from biological data is often problematic. In particular, without asymmetrical models of character change, root placement cannot be determined from the data alone and must rely on external assumptions (Kinene et al. 2016). Due to more favorable identifiability results that circumvent the need for root placement (Solís-Lemus and Ané 2016; Gross and Long 2018; Baños 2019; Gross et al. 2021), semi-directed networks have gained importance instead. However, their definition is somewhat problematic, in that it is given implicitly rather than explicitly. For instance, the mixed graph *G* in Fig. 1 is neither semi-directed nor multi-semi-directed, but how can this be decided? Of course, one possibility would be to develop some algorithm to make this decision, but for certain applications it could also be useful to have characterizations for when a mixed graph is semi-directed or multi-semi-directed.

In this paper, we shall provide some characterizations for semi-directed and multi-semi-directed networks. These results reveal combinatorial features of (multi-)semi-directed networks that help support further results in this paper and may also be helpful for future studies. One of the main tools that we use to obtain our characterizations is the concept of a *semi-directed cycle* in a mixed graph, that is, a cycle of arcs and edges in the graph whose edges can be oriented so as to obtain a directed cycle. Indeed, the exclusion of such cycles is a condition in one of our main characterizations (see e.g. Theorem 2). As a corollary of our characterizations, in case a mixed graph is a (multi-)semi-directed network, we give a more general characterization to that given in Maxfield et al. (2025) for when a subset of vertices or subdivisions of edges or arcs can correspond to a choice of root(s) that leads to a rooting of the network.

**Fig. 2:**
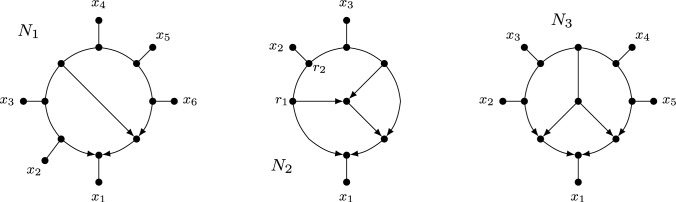
Three semi-directed networks $N_1$, $N_2$ and $N_3$. Semi-directed network $N_1$ is strongly tree-based since each of its rootings is tree-based. Semi-directed network $N_2$ is weakly tree-based, but not strongly, since the rooting obtained by directing all edges away from the vertex $r_1$ is tree-based, but the rooting obtained in a similar way using vertex $r_2$ is not. Semi-directed network $N_3$ is not weakly tree-based since it has no rooting that is tree-based

We shall also explore the consequences of our results for some special classes of rooted phylogenetic networks (see e.g. Kong et al. 2022 for a recent review of the different types of rooted networks). In particular, given a fixed class of rooted or multi-rooted phylogenetic networks, it is of interest to characterize when a (multi-)semi-directed network has a rooting contained in the given class. This could be either strongly (all rootings are in the class) or weakly (there exists a rooting that is in the class). For example, a rooted phylogenetic network is *tree-based* (Francis and Steel 2015) if it has a rooted spanning tree with the same leaf set as the network. Thus, a semi-directed network is *weakly tree-based* if it has a rooting that is tree-based, and it is *strongly tree-based* if all its rootings are tree-based (see e.g. Fig. 2).

Various concepts have been used to characterize when a rooted phylogenetic network is contained within a certain class. For example, the classes of rooted tree-child networks (Cardona et al. 2009) and rooted orchard networks (Erdos et al. 2019; Janssen and Murakami 2021) can both be characterized using cherry picking sequences (Erdos et al. 2019; Janssen and Murakami 2021), eventually leading to practical software for reconciling phylogenetic trees into networks (Bernardini et al. 2023, 2024). On the other hand, rooted tree-based networks (Francis and Steel 2015) have been characterized using both omnians (Jetten and van Iersel 2016) and path partitions (Francis et al. 2018), with the latter concept also providing a characterization for rooted forest-based networks (Huber et al. 2022). We shall generalize some of these concepts to (multi-)semi-directed networks and apply them to obtain characterizations for when such a network has a rooting that is contained within the classes of tree-child, orchard, tree-based or forest-based networks (or when all of its rootings are contained within these classes).

### Previous work

There is a well-established body of literature devoted to the study of rooted phylogenetic networks, with numerous structural classes receiving extensive attention (see again Kong et al. 2022). In parallel, undirected phylogenetic networks have also been studied when no information of directionality is available (see e.g. Gambette et al. 2012). This has led to work that focuses on the relationship between rooted and undirected networks. Recent contributions in this area include studies such as Huber et al. (2024); Maeda et al. (2023); van Iersel et al. (2018); Fischer and Francis (2020); Urata et al. (2024); Döcker and Linz (2024, 2025); Dempsey et al. (2024); Garvardt et al. (2023), which explore how undirected networks can be oriented and when this can be done to give rooted networks within specific classes.

In terms of (multi)-semi-directed networks, similar structural and graph-theoretical questions remain largely unexplored. Indeed, although seemingly related, the partial presence of directions within these networks presents fundamentally different problems. To date, the only substantial work in this direction is by Maxfield et al. (2025). The main aim of their work was to introduce an efficiently computable dissimilarity metric between two tree-child multi-semi-directed networks. To do this they developed some results for directing mixed graphs as multi-semi-directed networks. There are, however, several important differences between their framework and ours. Most notably, our definition of (multi-)semi-directed networks allows rootings on edges or arcs and we enforce arcs to correspond to reticulation arcs, whereas they do not, and they permit parallel edges or arcs, whereas we do not. For their type of networks they present two results that are related to ours: a characterization for when a subset of vertices in a mixed graph gives a rooting of the graph [(Maxfield et al. 2025), Proposition 8 and Remark 1], and when a mixed graph can be rooted to give a tree-child network in case such a rooting exists [(Maxfield et al. 2025), Proposition 11]. We give some more details on the relationship of these results with ours below.

### Outline of the paper

We now summarize the contents of the rest of this paper. After presenting some preliminaries in Sect. 2, in Sect. 3 we present some characterizations for when a mixed graph is multi-semi- and semi-directed (Theorems 1 & 2 and Corollary 1, respectively). Then in Sect. 4, we characterize the feasible sets of root locations in rootings of the network (Theorem 4). At the end of the section we also explain how our characterizations lead to an efficient algorithm for deciding if an arbitrary mixed graph is a multi-semi-directed or semi-directed network. In Sect. 5 we use omnians to characterize when a (multi)-semi-directed network is strongly and weakly tree-child (Theorem 5 and Proposition 1, respectively) and also strongly tree-based (Theorem 6). In Sect. 6 we use cherry picking sequences to characterize when a (multi)-semi-directed network is strongly or weakly orchard (Theorem 7 and Theorem 8, respectively), and in Sect. 7 we use path partitions to characterize when the network is weakly forest-based (Theorem 9) or weakly tree-based (Corollary 4). In Sect. 8, we conclude with some open problems.

## Preliminaries

### Mixed graphs

A *mixed graph* is an ordered tuple $G=(V,E,A)$ where *V* is a nonempty set of vertices, *E* is a set of undirected *edges* $\{u,v\}\subseteq V$, $u\ne v$, and *A* is a set of directed *arcs* (*u*, *v*) with $u,v\in V$, $u\ne v$, and such that for all arcs $(u,v)\in A$ we have that $\{u,v\}\notin E$ and $(v,u)\notin A$. Note that, by definition, parallel arcs, parallel edges or parallel edge/arc pairs are not allowed in mixed graphs. For an arc $(u,v)\in A$, we call *u* the *tail* and *v* the *head*. If $(u,v)\in A$, we call *u* a *parent* of *v* and *v* a *child* of *u*. If there is an edge $\{u,v\}\in E$ or an arc $(u,v)\in A$, we call *u* and *v* *adjacent* or *neighbours*.

Suppose for the following that $G=(V,E,A)$ is a mixed graph. For $v\in V$, the *indegree* $d_G^-(v)$ is the number of arcs entering *v*, the *outdegree* $d_G^+(v)$ is the number of arcs leaving *v*, and $d_G^e(v)$ is the number of edges in *E* incident to *v*. In addition, the *degree* $d_G(v)$ is the total number of edges and arcs incident to *v*. We will omit the subscript *G* when the graph is clear from the context. We call *v* a *reticulation* if $d^-(v)>1$, a *leaf* if $d(v)=d^e(v)=1$ or $d(v)=d^-(v)=1$ and a *root* if $d^+(v)=d(v)$. The set of leaves of *G* is called the *leaf set* of *G*, and it is denoted by *L*(*G*). We say that *G* is *binary* if $d(v)\in \{1,2\}$ for each root $v\in V$ and $d(v)\in \{1,3\}$ for each non-root $v\in V$.

A *path* in *G* is a sequence of pairwise distinct vertices $(v_1,\ldots ,v_p)$, $p \ge 1$, such that for all $i\in \{1,\ldots ,p-1\}$ either $(v_i,v_{i+1})$ or $(v_{i+1},v_{i})$ is an arc in *A* or $\{v_i,v_{i+1}\}$ is an edge in *E*. Such a sequence is a *semi-directed path* (from $v_1$ to $v_p$) if for all $i\in \{1,\ldots ,p-1\}$ either $(v_i,v_{i+1})$ is an arc in *A* or $\{v_i,v_{i+1}\}$ is an edge in *E*. A $\wedge$*-path* (between $v_1$ and $v_p$) in *G* is a path $(v_1,\ldots ,v_i,\ldots ,v_p)$ of *N*, $p \ge 1$, such that $(v_i,\ldots ,v_1)$ and $(v_i,\ldots ,v_p)$ are semi-directed paths, for some $i\in \{1,\ldots ,p\}$. An *edge-path* in *G* is a path $(v_1,\ldots ,v_p)$, such that $\{v_i,v_{i+1}\}$ is an edge in *E*, for all $i\in \{1,\ldots ,p-1\}$. We call the number of vertices on a path *P* minus 1 the *length* of *P* and refer to *P* as a *trivial* path if the length of *P* is zero. If *P* is not trivial then we sometimes also say that *P* is *non-trivial*. We say that *G* is *connected* if for any two vertices *x* and *y* of *G* there is a path joining *x* and *y*

**Fig. 3:**
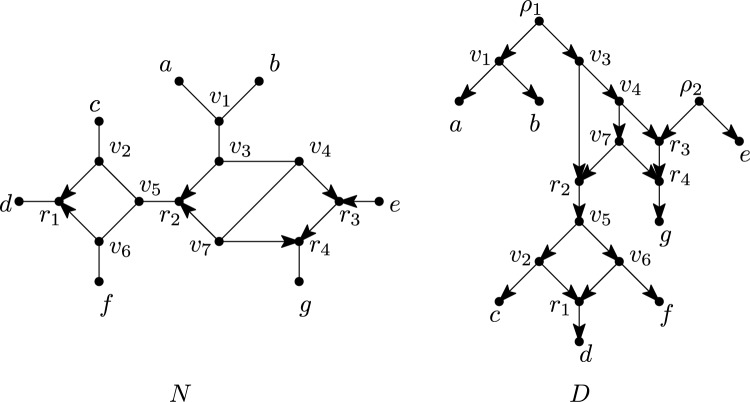
*Left:* A 2-semi-directed network *N* on $\{a,b,\ldots , e\}$ with set of reticulations $\{r_1,r_2,r_3,r_4\}$. The sequence $(r_1,v_2,v_5,r_2,v_3,v_4,r_3,r_4,g)$ is a $\wedge$-path of *N* and the sequence $C=(r_2,v_3,v_4,r_3,r_4,v_7,{r_2})$ is a cycle of *N*. The vertices $r_2$ and $r_4$ are sinks of *C* whereas $r_3$ is not. The path $(a,v_1,v_3,v_4,v_7)$ is an example of an edge-path. The leaves *a* and *b* form a cherry, leaves *d* and *g* are reticulation leaves, while $r_1$ and $r_4$ are leaf reticulations. The vertex sets of the source components are $\{a,b,v_1,v_3,v_4,v_7\}$ and $\{e\}$ while the sink components have vertex sets $\{d,r_1\}$ and $\{g,r_4\}$. *Right:* A rooting *D* of *N* in the form of a 2-rooted network with roots $\rho _1$ and $\rho _2$

A *cycle* in *G* is a sequence of vertices $(v_1,v_2,\ldots ,v_p=v_1)$, $p \ge 4$, such that $v_i\ne v_j$ for $1\le i<j<p$ and, for all $i\in \{1,\ldots ,p-1\}$, either $(v_i,v_{i+1})$ or $(v_{i+1},v_{i})$ is an arc in *A* or $\{v_i,v_{i+1}\}$ is an edge in *E*. Note that, since $p\ge 4$ and $v_p=v_1$, a cycle contains at least three distinct vertices. A cycle is called *semi-directed* if, for all $i\in \{1,\ldots ,p-1\}$, either $(v_i,v_{i+1})$ is an arc in *A* or $\{v_i,v_{i+1}\}$ is an edge in *E*. A reticulation *r* of *N* is a *sink* of a cycle in *N* if $r=v_i$ and $(v_{i-1},v_i),(v_{i+1},v_i)\in A$, for some $i\in \{1,\ldots , p-1\}$, with $v_0=v_{p-1}$. If *G* is connected and does not contain a cycle, then we call *G* a *tree*. If additionally $E = \emptyset$, i.e., *G* is fully directed, and *G* has a single root, we call *G* a *rooted tree*. We refer to Fig. 3 for examples that illustrate some of the definitions in this and the next subsection.

### Multi-rooted and multi-semi-directed networks

Suppose *X* is a finite set with at least two elements. A *multi-rooted network (on X)* is a mixed graph (*V*, *E*, *A*) (with leaf set *X*), with $E=\emptyset$, no directed cycles, $d(v)\ne 2$ for all non-root vertices $v\in V$ and $d^-(v)\in \{0,{1}, d(v)-1\}$ for all $v\in V$. A *k-rooted network* is a multi-rooted network with precisely $k\ge 1$ roots. A 1-rooted network is also called a *rooted network*. A rooted network without any reticulations is called a *rooted phylogenetic tree*.

Consider any mixed graph. *Subdividing* an edge $\{u,v\}$ replaces the edge $\{u,v\}$ by two edges $\{u,w\}$ and $\{w,v\}$ with *w* a new vertex. *Subdividing* an arc (*u*, *v*) replaces the arc (*u*, *v*) by an edge $\{u,w\}$ and an arc (*w*, *v*) with *w* a new vertex. *Suppressing* a degree-2 vertex *w* is defined as follows:

- if *w* has two incident edges $\{u,w\},\{w,v\}$, replace them by a single edge $\{u,v\}$;
- if *w* has two incident arcs (*u*, *w*), (*w*, *v*), replace them by a single arc (*u*, *v*);
- if *w* has an incident edge $\{u,w\}$ and an incident arc (*w*, *v*), replace them by a single arc (*u*, *v*);
- if *w* has an incident arc (*u*, *w*) and an incident edge $\{w,v\}$, replace them by a single edge $\{u,v\}$,

and in each of these cases also delete *w*. Note that degree-2 vertices with two incoming or two outgoing arcs are not suppressed.

The *semi-deorientation* of a multi-rooted network *D* is the result of replacing each arc (*u*, *v*) of *D* by an edge $\{u,v\}$ if $d_D^-(v)=1$ and afterwards suppressing any vertex $\rho$ with $d_D(\rho )=2$. Note that a root with two outgoing arcs in *D* will still be present in the semi-deorientation. Also note that a semi-deorientation is not necessarily a mixed graph since suppressing roots may lead to parallel arcs. However, we will only consider mixed graphs in this paper and hence not consider cases with parallel arcs. A *rooting*^1^ of a mixed graph *G* is a multi-rooted network *D* such that *G* is the semi-deorientation of *D*. Observe that *D* can be obtained from *G* by subdividing (zero or more) arcs and/or edges and replacing edges by arcs. Note that the subdivision vertices necessarily become roots in *D* and that vertices of *G* may also become roots in *D*. This also includes the possibility that a root of *G* that is contained in *X* becomes a leaf in *D* (see e.g. the vertex *e* in Fig. 3) or that a leaf of *G* not contained in *X* becomes an outdegree-1 root in *D*^2^. However, it is not possible to create any new reticulations. A *k-semi-directed network* (on *X*) is a mixed graph that is the semi-deorientation of some *k*-rooted network (with leaf set *X*). Note that the leaf set of a *k*-semi-directed network on *X* may not be equal to *X*, because an outdegree-1 root may also be in *X* (see, for example, the vertex *e* in Fig. 3 again). A *multi-semi-directed network* is a *k*-semi-directed network for some $k\ge 1$. A *semi-directed network* is a 1-semi-directed network. When drawing multi-rooted and multi-semi-directed networks, we will often omit the leaf labels when they are not relevant. We will reserve the letter *G* for general mixed graphs, *D* for (multi)-rooted networks and *N* for (multi)-semi-directed networks.

## Characterizations of multi-semi-directed networks

In this section we characterize when a mixed graph is a multi-semi-directed network or when it is in fact a semi-directed network. These characterizations naturally lead to algorithms for checking (multi-)semi-directedness. We sketch one efficient approach at the end of Sect. 4, which outlines a method based on rootings of (multi-)semi-directed networks.

Suppose $G=(V,E,A)$ is a mixed graph. A *cherry* of a mixed graph is an ordered pair of leaves (*x*, *y*) such that there is a length-2 path between *x* and *y*, either consisting of two edges or of two arcs directed towards *x* and *y*, respectively. For convenience, we also refer to a cherry with leaves *x* and *y* as a cherry on $\{x,y\}$. A *leaf reticulation* of *G* is a reticulation of *G* that is adjacent to a leaf of *G* and a *reticulation leaf* of *G* is a leaf of *G* that is adjacent to a reticulation of *G*.

An *(undirected) sink component* of $G=(V,E,A)$ is a connected component *C* of the (undirected) graph (*V*, *E*) such that there are no arcs $(u,v)\in A$ with $u\in C$ and $v\notin C$. For example, in Fig. 3, $\{d,r_1\}$ is a sink component since it is a connected component of the graph that is obtained by ignoring all arcs, and in *G* there are no arcs leaving this component. Similarly, an *(undirected) source component*^3^ of *G* is a connected component *C* of (*V*, *E*) such that there are no arcs $(u,v)\in A$ with $u\notin C$ and $v\in C$. A subgraph $G'$ of *G* is a *pendant subtree* if $G'$ is a tree and has at most one vertex that has a neighbour in *G* not in $G'$.

We start with the following technical result which we use to prove our first characterization of multi-semi-directed networks.

### Lemma 1

Suppose $G=(V,E,A)$ is a connected mixed graph with $|V|\ge 3$. Then *G* contains either a cherry or a leaf reticulation (or both) if the following properties hold:

(Ci) $d(v)\ne 2$ and $d^-(v)\in \{0,d(v)-1\}$ for all $v\in V$;
(Cii) each cycle of *G* contains at least one sink; and
(Ciii) each sink component of *G* is a pendant subtree.

### Proof

Let *P* be a semi-directed path in *G* containing a maximum number of arcs and, over all such paths, containing a maximum number of edges. If *P* contains no arc, then it follows that $A=\emptyset$ and hence *G* is a tree by (Cii) and since *G* is connected. Since *G* has at least three vertices, and no degree-2 vertices, it follows that *G* has a cherry. Therefore, we may assume that *P* contains at least one arc. Let *s* denote the first vertex on *P*.

We next show that *P* ends in a leaf or in a reticulation. To see this, assume for contradiction that *P* ends in a vertex *v* with $d(v)\ne 1$ and $d^-(v)=0$. Then there is an edge $\{v,w\}$ or an arc (*v*, *w*) with *w* not on *P* (since otherwise we would have a cycle without sink, which is not allowed by (Cii)). Hence, we can extend *P* to a semi-directed path containing more arcs or the same number of arcs and more edges, by appending *w* in contradiction to the maximality of *P*. Thus, *P* ends in a leaf or in a reticulation, as required.

To complete the proof, we next show that *P* ends in a reticulation leaf or in a leaf that is in a cherry. To prove this, assume that *P* ends in a reticulation or in a leaf that is not a reticulation leaf and not in a cherry. First suppose that *P* ends in a leaf *x* that is not a reticulation leaf. Then, in view of (Ci), there exists a vertex *z* of *N* that is not on *P* and, denoting by *w* the vertex on *P* that is the predecessor of *v* on *P*, we have that $\{w,z\}\in E$ or $(w,z)\in A$. Hence, *z* is either also a leaf or it is a reticulation. In the first case, *x* is in a cherry. Therefore, we may and will assume that *P* ends in a reticulation *v*. Furthermore, there exist arcs entering *v* that are not on *P* and, by the maximality of *P*, the last edge/arc of *P* is an edge.

Consider the maximal connected subgraph *H* of *G* containing *v* but no arcs. Observe that *H* is not a pendant subtree since it contains at least two vertices with incoming arcs (*v* and the head of the last arc of *P*). Hence, by (Ciii), *H* is not a sink component. It follows that *H* has a vertex *y* with an incident outgoing arc $a=(y,w)$, some $w\in V$. Hence, the subpath of *P* from *s* to *y* extended by *w* has one more arc than *P* contradicting the assumption that *P* contains a maximum number of arcs. $\square$

### Theorem 1

A mixed graph $G=(V,E,A)$ is a multi-semi-directed network if and only if Properties (Ci) - (Ciii) hold.

### Proof

If *G* is a multi-semi-directed network, then Properties (Ci) - (Ciii) clearly hold.

To see the converse, assume that Properties (Ci) - (Ciii) hold. We perform induction on $|A|+|E|$. The base case is $|A|+|E|=0$. In this case, *G* is a set of isolated vertices. Hence, *G* is its own semi-deorientation. Thus, *G* is a multi-semi-directed network.

For the inductive step, assume that *G* is such that $|A|+|E|\ge 1$. Then *G* must contain a connected component that is not an isolated vertex. In view of the base case it suffices to show that every connected component of *G* that is not an isolated vertex is a multi-semi-directed network. Let $G'$ be a connected component of *G* that is not an isolated vertex. If $G'$ has exactly two vertices, then they are connected by an edge by (Ci) and clearly $G'$ is (multi-)semi-directed. Otherwise, by Lemma 1, $G'$ must contains either a cherry or a leaf reticulation (or both).

Assume first that $G'$ contains a cherry on $\{x,y\}$. Then, by (Ci), the length-2 path between *x* and *y* consists of two edges. In this case, we delete leaf *x* from this cherry and suppress the vertex adjacent to *x* if this has rendered it a vertex of degree two. The resulting graph $G''$ is a multi-semi-directed network by induction. This means that $G''$ is the semi-deorientation of a multi-rooted network $D''$. Let $D'$ be obtained from $D''$ by subdividing the arc entering *y* by a new vertex *p* and adding leaf *x* with an arc (*p*, *x*). Then $D'$ is a multi-rooted network and the semi-deorientation of $D'$ is $G'$. Hence, $G'$ is a multi-semi-directed network.

To conclude the proof, assume that $G'$ contains a reticulation leaf *z*. Let *r* be the reticulation adjacent to *z* and let $p_1,\ldots ,p_{t}$ be the parents of *r* (with $t=d^-(r)$). Let $G''$ be obtained from $G'$ by replacing vertices *r*, *z* by vertices $z_1,\ldots , z_t$ and replacing arc $(p_i,r)$ by an edge $\{p_i,z_i\}$ for $i\in \{1,\ldots ,t\}$ and deleting edge $\{r,z\}$ thus reducing $|A|+|E|$ by 1. The resulting graph $G''$ is a multi-semi-directed network by induction. This means that $G''$ is the semi-deorientation of a multi-rooted network $D''$. Let $D'$ be obtained from $D''$ by merging $z_1,\ldots ,z_t$ into a single vertex *r* and adding a leaf *z* with an arc (*r*, *z*). Then, $D'$ is a multi-rooted network and the semi-deorientation of $D'$ is $G'$. Hence, $G'$ is a multi-semi-directed network. $\square$

The following lemma can be used to show an alternative characterization of multi-semi-directed networks.

### Lemma 2

Let *C* be a cycle in a mixed graph *G*. If

- *C* is not semi-directed; and
- *C* contains no non-trivial edge-path between two reticulations of *G*,

then *C* contains at least one sink.

### Proof

Let $C=(v_1,v_2,\ldots ,v_p=v_1)$, $p\ge 4$. If *C* is not semi-directed, then there exist $i,j\in \{1,2,\ldots , p-1\}$ distinct such that $(v_i,v_{i+1})$ and $(v_{j+1},v_j)$ are arcs on *C*. Without loss of generality, $i<j$ $(\textrm{mod}\,\,\,\,p-1)$. Assume that *j* and *i* are such that $j-i$ is minimized. If $j-i=1$, then *C* contains the sink $v_j=v_{i+1}$. Otherwise, $j-i>1$ and, so, $v_{i+1}$ and $v_j$ are both reticulations of *G* and *C* contains a non-trivial edge-path between them. $\square$

**Fig. 4:**
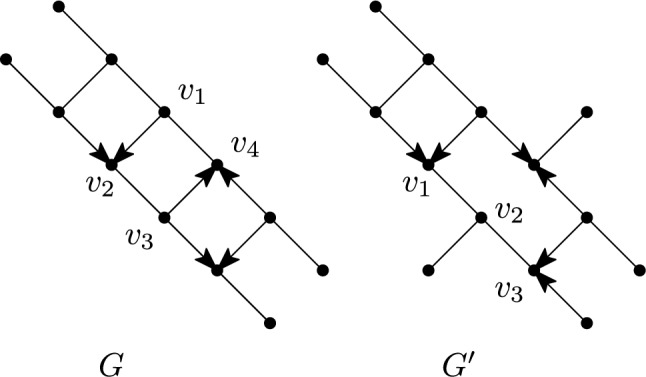
Two mixed graphs *G* and $G'$ that, by Theorem 2, are not multi-semi-directed networks. The reason is that *G* contains the semi-directed cycle $(v_1,v_2,v_3,v_4,v_1)$ while $G'$ contains the non-trivial edge-path $(v_1,v_2,v_3)$ and $v_1$ and $v_3$ are reticulations

An alternative characterization of mixed graphs that are multi-semi-directed networks is as follows. See Fig. 4 for examples that illustrate that Properties (II) and (III) cannot be weakened.

### Theorem 2

A mixed graph $G=(V,E,A)$ is a multi-semi-directed network if and only if

(I) $d(v)\ne 2$ and $d^-(v)\in \{0,d(v)-1\}$ for all $v\in V$;
(II) *G* contains no semi-directed cycle; and
(III) *G* contains no non-trivial edge-path between two reticulations.

### Proof

As in the case of Theorem 1, it is straight-forward to see that if *G* is a multi-semi-directed network, then Properties (I), (II) and (III) hold.

For the converse direction, by Theorem 1, it suffices to show that Properties (I), (II) and (III) imply Properties (Ci), (Cii) and (Ciii). For this, it suffices to show that Properties (II) and (III) imply Properties (Cii) and (Ciii).

First observe that Property (III) implies Property (Ciii) because any sink component that is not a pendant subtree contains two reticulations and a non-trivial edge-path path joining them. In view of Lemma 2, Properties (II) and (III) together imply Property (Cii). $\square$

**Fig. 5:**
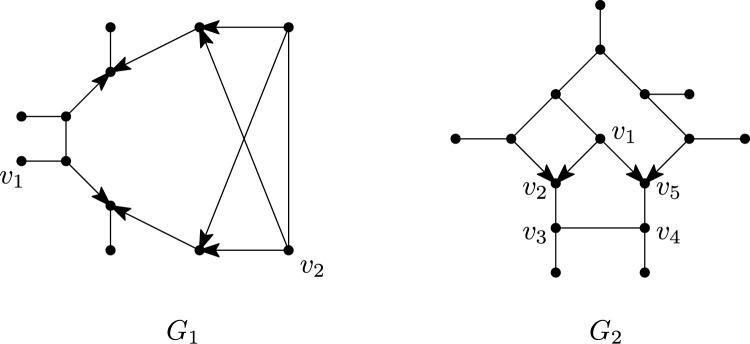
*Left:* A mixed graph $G_1$ which, by Corollary 1, is not a semi-directed network since there is no $\wedge$-path between $v_1$ and $v_2$. *Right:* A mixed graph $G_2$ that, by Corollary 1, is not a semi-directed network since it contains a cycle $(v_1,v_2,v_3,v_4,v_5,v_1)$ without a sink

We will now characterize semi-directed networks. See Fig. 5 for examples that illustrate that Properties (2) and (3) cannot be weakened. Recall that a semi-directed network is the semi-deorientation of some 1-rooted network, i.e. a rooted network with a single root.

### Corollary 1

A mixed graph $G=(V,E,A)$ is a semi-directed network if and only if

1. $d(v)\ne 2$ and $d^-(v)\in \{0,d(v)-1\}$ for all $v\in V$;
2. each cycle of *G* contains at least one sink; and
3. *G* contains a $\wedge$-path between each pair of vertices $u,v\in V$.

### Proof

As before, it is easy to see that if *G* is a semi-directed network then Properties (1), (2) and (3) hold.

Now assume that Properties (1), (2) and (3) hold. We first show that *G* is a multi-semi-directed network. In view of Theorem 1, it suffices to show that Properties (1) - (3) imply Property (Ciii). To this end, suppose that *G* contains a sink component *S* that is not a pendant subtree. Note that *S* is a tree by Property (2). Since *S* is not pendant, it contains at least two reticulations $r_1,r_2$. Let $p_1,p_2$ be parents of $r_1,r_2$, respectively, such that $p_1\ne p_2$. Note that $p_1,p_2$ are not in *S*. By Property (3), there exists a $\wedge$-path *P* between $p_1$ and $p_2$. Hence, there is a cycle without sink, formed by path *P* together with the arcs $(p_1,r_1),(p_2,r_2)$ and the path between $r_1$ and $r_2$ through *S*, which is a contradiction to (2). Thus, (Ciii) holds, as required.

It remains to show that any rooting of *G* has a single root. Suppose *D* is a rooting of *G* with at least two roots $\rho _1,\rho _2$. Then there is no $\wedge$-path between $\rho _1$ and $\rho _2$ in *D* and hence also not in *G*, contradicting Property (3). Hence, we can conclude that *G* is a semi-directed network. $\square$

## Rootings of multi-semi-directed networks

The following theorem and its corollary show that the number of roots of a rooting of a multi-semi-directed network $N=(V,E,A)$ can be calculated directly from the *reticulation number* $|A|-|R|$ of *N*, with *R* the set of reticulations.

### Theorem 3

If $N=(V,E,A)$ is a k-semi-directed network, some $k\ge 1$, then the reticulation number of *N* equals

$$|E|+|A|-|V|+k.$$

### Proof

Consider the graph *F* obtained from *N* by deleting all arcs. Then *F* is a forest since, by Theorem 2, *N* does not contain any cycles traversing only edges. In any rooting *D* of *N*, where we root only on vertices of *N*, each (connected) component *T* of *F* is oriented as a rooted tree $T^r$. The root of $T^r$ is either a root of *D* or a reticulation of *D*. Moreover, each vertex of $T^r$ that is not the root of $T^r$ is not a reticulation of *D* and also not a root of *D*. Hence, each component of *F* contains exactly one vertex that is a root or a reticulation of *D*. It follows that the number of components of *F* is precisely $|R|+k$ where *R* is the set of reticulations of *N*.

In any forest, the number of components equals the number of vertices minus the number of edges. Therefore,

$$|V|-|E| = |R|+k,$$

implying that

$$|A| + |V|-|E| = |A|+|R|+k,$$

which can be rewritten as

$$|A| - |R| = |E| + |A| -|V|+ k.$$

Since $|A|-|R|$ is the reticulation number of *N*, the theorem follows. $\square$

As an immediate consequence, we have the following result.

### Corollary 2

If $N=(V,E,A)$ is a multi-semi-directed network, then all rootings of *N* have

$$|V|-|R|-|E|$$

roots, with *R* the set of reticulations of *N*.

We now prove an auxiliary result that will be useful to characterize all possible rootings of a multi-semi-directed network.

### Lemma 3

Let *N* be a multi-semi-directed network, *R* the set of reticulations of *N*, and $U\subseteq V(N)$ such that there is no edge-path between any two vertices in $U\cup R$. Then there exists a rooting of *N* in which each vertex of *U* is a root.

### Proof

The proof is by induction on |*U*|. The base case for $U=\emptyset$ is trivial. If $|U|\ge 1$, consider a vertex $u\in U$. By induction, there exists a rooting *D* of *N* in which each vertex of $U\setminus \{u\}$ is a root. Let $C_u$ be the connected component of the graph obtained from *N* by deleting all arcs, such that $C_u$ contains *u*. First observe that *u* is the only vertex from *U* in $C_u$ since otherwise there would be an edge-path between two vertices in *U*. Also note that $C_u$ is a source component since there is no edge-path between *u* and any reticulation. Moreover, by Theorem 2, *N* contains no cycle consisting of only edges. Hence, $C_u$ is a tree. Since no vertex of $C_u$ is a reticulation in $C_u$, it follows that $C_u$ is a rooted tree in *D*. The root $u'$ of this tree is also a root of *D*, since *D* contains no arcs whose head is a vertex in $C_u$ but whose tail is not. Hence, in *D*, $C_u$ contains exactly one root $u'$. If $u'=u$ then $u'\in U$ and *D* is a rooting of *N* in which $u'$ is a root. Hence, the lemma holds in this case.

If $u'\ne u$ then, since $C_u$ is a rooted tree in *D*, we can modify the orientation of *D* to obtain an alternative rooting of *N* by changing only the orientation of each arc on the path between *u* and $u'$ in $C_u$. This gives a rooting $D'$ of *N* in which $u\in U$ is a root. Moreover, all vertices in $U\setminus \{u\}$ are still roots of $D'$, which concludes the proof. $\square$

For a rooting *D* of multi-semi-directed network $N=(V,E,A)$, define the *root configuration* as the triple $(V',E',A')$ with $V'\subseteq V$, $E'\subseteq E$ and $A'\subseteq A$ such that the roots of *D* are precisely the vertices in $V'$ together with vertices subdividing each edge in $E'$ and arc in $A'$. In the following theorem, we characterize the valid root configurations of a multi-semi-directed network. As noted in the introduction, a similar characterization was given in [(Maxfield et al. 2025), Proposition 8], although it was under a slightly different framework (e.g. assuming $A' = E' = \emptyset$).

### Theorem 4

Let $N=(V,E,A)$ be a multi-semi-directed network and $V'\subseteq V$, $E'\subseteq E$ and $A'\subseteq A$. Then there exists a rooting *D* of *N* with root configuration $(V',E',A')$ if and only if

- each vertex in $V'$ and each edge in $E'$ is in a source component of *N* and each arc in $A'$ is an outgoing arc of a source component of *N*; and
- each source component of *N* contains exactly one element of $V'$, contains exactly one edge in $E'$ or has exactly one outgoing arc in $A'$.

### Proof

We first prove the “only if” direction. Since *N* is multi-semi-directed, there exists a rooting *D* of *N*. Let $(V'',E'',A'')$ be its root configuration.

First suppose that there is a $v\in V''$ that is not in a source component of *N*. Then there exists a reticulation *r* in the component *C* of the graph (*V*, *E*) containing *v* with both incoming arcs of *r* having their tail outside *C*. Note that $v\ne r$. Hence, there exists an edge-path between *r* and *v* in *N*. Since *v* is a root of *D*, this path is a directed path from *v* to *r* in *D*. However, this implies that $d_D^-(r)=d_D(r)$, contradicting the definition of a multi-rooted network. The other cases (that there is a $e\in E''$ that is not in a source component or an arc $a\in A''$ that is not an outgoing arc of a source component) are handled similarly.

Now suppose that there exists a source component *C* of *N* containing $v,w\in V''$ with $v\ne w$. Then there exists an edge-path between *v* and *w* of length at least 2 in *N*. Since *v* is a root in *D*, this path is directed from *v* to *w* in *D*. However, since *w* is also a root in *D*, the path is directed from *w* to *v* in *D*, a contradiction. The other cases are again handled similarly.

We now prove the “if” direction. Since *N* is multi-semi-directed, there exists a rooting *D* of *N*. Let $(V'',E'',A'')$ be its root configuration.

For an edge $\{u,v\}$ of *N*, clearly, there exists a rooting of *N* with root *u* if and only if there exists a rooting with root *w* subdividing $\{u,v\}$. Similarly, for an arc (*u*, *v*) of *N*, clearly, there exists a rooting of *N* with root *u* if and only if there exists a rooting with root *w* subdividing (*u*, *v*). Hence, we may assume $E'=A'=E''=A''=\emptyset$. Let *R* be the set of reticulations of *N*. Since each element of $V'$ is in a different source component, there is no edge-path between any two vertices in $R\cup V'$. Then, by Lemma 3, it follows that there exists a rooting of *N* with root configuration $(V',E',A')$. $\square$

Theorem 4 directly leads to an efficient algorithm taking $O(|V| + |E| + |A|)$ time for deciding if an arbitrary mixed graph $G = (V, E, A)$ is a (multi-)semi-directed network (note that in [(Maxfield et al. 2025), Remark 1] a similar algorithm is sketched to efficiently find a rooting of a multi-semi-directed network). First, find all the source components of *G* by traversing the graph in $O(|V| + |E| + |A|)$ time. Second, in each source component, pick an arbitrary vertex as root. Third, do a breath-first search from each root and orient all edges away from the root, again taking $O(|V| + |E| + |A|)$ time. Finally, with yet another traversal, check if the resulting mixed graph $G'$ is a multi-rooted directed network and whether its semi-deorientation is *G*. Note that by additionally checking whether or not $G'$ has a single root, it can also be determined if *G* is semi-directed.

## Omnians

In this section, we show how the concept of omnians can be used to characterize tree-child (Theorem 5 and Proposition 1) and tree-based multi-semi-directed networks (Theorem 6). As remarked in the introduction, [(Maxfield et al. 2025), Proposition 11] gives an alternative characterization for the tree-child case where, instead of using omnians the concept of the “directed part” of a network is used.

**Fig. 6:**
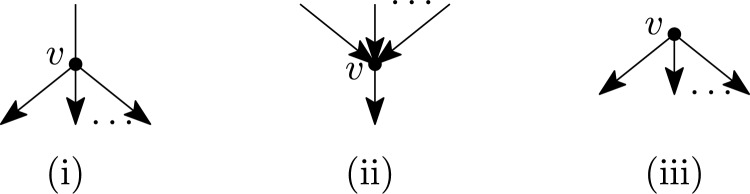
The three possible configurations surrounding an omnian in a multi-semi-directed network: (i) one incident edge and at least two outgoing arcs, (ii) at least two incoming arcs and one outgoing arc and (iii) at least two outgoing arcs (and in all cases no other incident edge/arcs). Note that Case (iii) is possible in multi-semi-directed networks but not in semi-directed networks

**Fig. 7:**
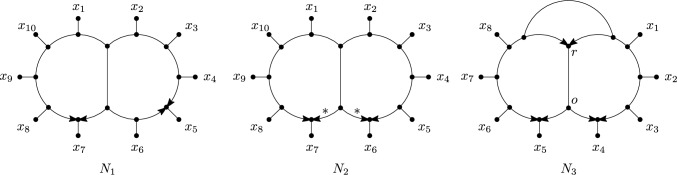
The semi-directed network $N_1$ on $X=\{x_1,\ldots , x_{10}\}$ is strongly tree-child in view of Theorem 5. The semi-directed network $N_2$ also on *X* is weakly tree-child, but not strongly, since the rootings with the root subdividing an arc labelled $*$ are tree-child, but all other rootings are not tree-child (see Corollary 3). The semi-directed network $N_3$ on $\{x_1\ldots , x_8 \}$ is not weakly tree-child since the edge $\{r,o\}$ forms an edge-path between a reticulation marked *r* and an omnian marked *o* (see Corollary 3)

The main definitions of this section are the following. We say that a vertex *v* of a multi-semi-directed network is an *omnian* if $d^+(v)\ge 1$ and $d^e(v)\le 1$, see Fig. 6. We call a multi-rooted network *D* *tree-child* if each non-leaf vertex of *D* has at least one child that is not a reticulation. Furthermore, we say that a multi-semi-directed network *N* is *weakly tree-child* if *N* has a rooting that is tree-child. In this case, we also call such a rooting of *N* a *tree-child rooting of N.* Finally, we say that *N* is *strongly tree-child* if every rooting of *N* is tree-child. See Fig. 7 for examples.

### Theorem 5

A multi-semi-directed network *N* is strongly tree-child if and only if *N* has no omnians.

### Proof

First assume that *N* is strongly tree-child. Suppose that there exists a vertex $v\in V$ that is an omnian, i.e., $d^+(v)\ge 1$ and $d^e(v)\le 1$. If $d^e(v)=0$, then all children of *v* are reticulations in any rooting of *N* and *v* is not a leaf since $d^+(v)> 0$. This would contradict that *N* is strongly tree-child. Hence, $d^e(v)=1$. By Theorem 1 (Property (Ci)), $d^-(v)\in \{0,d(v)-1\}$. Since $d^-(v)=d(v)-d^+(v)-d^e(v) \le d(v) -1 - 1$, this means that $d^-(v)=0$ and thus $d^+(v)=d(v)-1$.

Let $e=\{u,v\}$ be the edge in *N* incident to *v*. Observe that all neighbours of *v* other than *u* are reticulations. Let *D* be a tree-child rooting of *N*. Since *v* has at least one child in *D* that is not a reticulation, the edge *e* is oriented away from *v* in *D*. This means that there are two possibilities. Either *v* is a root of *D*, or there exists a child *a* of *v* in *N* such that the arc (*v*, *a*) of *N* leaving *v* has been subdivided by a root *w* in *D* and *D* contains the arcs (*w*, *v*) and (*w*, *a*). In the first case, changing the orientation of the arc (*v*, *u*) to (*u*, *v*) (making *u* a root) gives a rooting $D'$ of *N* that is not tree-child. Also in the second case, $D'$ is a rooting of *N* that is not tree-child. In both cases, we obtain a contradiction, completing the first direction of the proof.

For the converse, suppose that *N* has no omnians, that is, $d^e(v)\ge 2$ or $d^+(v)=0$ for all $v\in V$. Assume that *N* is not strongly tree-child, i.e., there exists a rooting *D* of *N* and a non-leaf vertex *v* of *D* such that all children of *v* in *D* are reticulations. If *v* is a reticulation too, then $d^e_N(v)=0$ and $d^+_N(v)=1$. If *v* is not a reticulation, then $d^e_N(v)\le 1$ and $d^+_N(v)\ge 2$. In both cases, we obtain a contradiction. $\square$

### Proposition 1

Let $N=(V,E,A)$ be a *k*-semi-directed network, some $k\ge 1$, with set of reticulations *R* and set of omnians *O*. Then, *N* is weakly tree-child if and only if $|O| \le k$, $d^e(v)\ge 1$ for all $v\in V$, and there does not exist a non-trivial edge-path between any two vertices in $O\cup R$.

### Proof

Suppose that *N* is weakly tree-child. Then there exists a tree-child rooting *D* of *N*. For each vertex $u\in O$, either *u* is a root in *D* or one of the outgoing arcs of *u* is subdivided by a vertex that is a root in *D*. Hence, $|O|\le k$. For the second condition, assume for contradiction that there exists a vertex $v\in V$ with $d_N^e(v) = 0$. Then, by Theorem 2, $d_N^+(v) \ge 1$ and in any rooting *D* of *N*, every child of *v* is a reticulation. Thus, *D* is not tree-child; a contradiction. Lastly, to prove the third condition, assume for contradiction that there exists a non-trivial edge-path *P* in *N* between two vertices in $O\cup R$, say between *s* and *t*. In a tree-child rooting, an edge incident to a reticulation must be oriented away from the reticulation and, similarly, an edge incident to an omnian must be oriented away from the omnian. Hence, the edge of *P* incident to *s* is oriented away from *s* in *D*. Similarly, the edge of *P* incident to *t* is oriented away from *t* in *D*. Therefore, *P* must contain a reticulation in *D* and hence in *N*; a contradiction since no internal vertex of an edge-path can be a reticulation.

For the other implication, suppose that $|O|\le k$, that $d^e(v)\ge 1$ for all $v\in V$, and that there does not exist a non-trivial edge-path between any two vertices in $O\cup R$. By Lemma 3, there exists a rooting *D* of *N* such that each vertex in *O* is a root in *D*. Suppose that *D* is not tree-child. Let *v* be a non-leaf vertex of *D* all whose children in *D* are reticulations. Then, we obtain a contradiction since then either $d^e_N(v)=0$ or *v* is an omnian of *N* that is not a root of *D*. $\square$

We can simplify the characterization in Proposition 1 for semi-directed networks as follows, using that, in a (multi-)semi-directed network, $d^e (v) \ge 1$ holds for any non-omnian vertex *v* and there is no non-trivial edge-path between two reticulations (Theorem 2).

### Corollary 3

A semi-directed network *N* is weakly tree-child if and only if *N* has at most one omnian and if there is an omnian *o*, then $d^e (o) \ge 1$ and there is no non-trivial edge-path between *o* and a reticulation *r* of *N*.

**Fig. 8:**
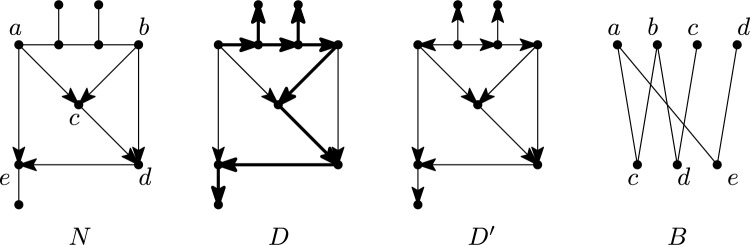
A semi-directed network *N* that is weakly tree-based but not strongly tree-based, along with a rooting *D* for it that is tree-based (a spanning tree with leaf set *L*(*D*) is indicated in bold) and a rooting $D'$ for it that is not tree-based. *Far right:* The bipartite graph *B* used in the proof of Theorem 6

We next turn our attention to tree-based multi-semi-directed networks. A *spanning tree* of a mixed graph *G* is a subtree of *G* that contains all vertices of *G* and is a tree. A multi-rooted network *N* is *tree-based* if it has a rooted spanning tree that has the same leaf set as *N*. Clearly, a multi-rooted network that is tree-based must be a rooted network, since the corresponding spanning tree must have a single root. Note that binary rooted tree-based networks were introduced in Francis and Steel (2015) and extended to non-binary rooted networks in Jetten and van Iersel (2016), who also defined the stricter notion of strictly tree-basedness, which we do not consider here. A multi-semi-directed network *N* is *weakly tree-based* if *N* has a rooting that is tree-based and *N* is *strongly tree-based* if every rooting of *N* is tree-based, see Fig. 8 for an example. Since every multi-rooted tree-based network is a rooted network, all multi-semi-directed networks that are weakly or strongly tree-based are semi-directed networks. Hence, we will focus on semi-directed networks for the remainder of this section.

To state our next result, we define for a multi-semi-directed network $N=(V,E,A)$ and a subset *S* of the set of omnians of *N* the set

$$\delta ^+(S):=\{t\in V\mid (s,t)\in A \text{ for } \text{ some } s\in S\}.$$

Hence, $\delta ^+(S)$ contains the vertices of *N* that are the head of an arc that starts at an omnian contained in *S*. Note that by definition, $\delta ^+ (S)$ may contain vertices that are also in *S*.

### Theorem 6

Let *N* be a semi-directed network with *O* its set of omnians. Then *N* is strongly tree-based if and only if for each $S\subseteq O$ we have that $|\delta ^+(S)|\ge |S|$.

### Proof

Let *R* be the set of reticulations of *N*. We follow a similar approach as in Jetten and van Iersel (2016) for directed networks. More precisely, we first associate a bipartite graph $B=(V',E')$ to *N* that has vertex set $V'=R'\cup O'$, with $R'$ containing a copy of each reticulation in *R* and $O'$ containing a copy of each omnian in *O*. Hence, a vertex of *N* that is a reticulation as well as an omnian has two corresponding vertices in $V'$. For $r\in R'$ and $o\in O'$, we define $\{r,o\}$ to be an edge in $E'$ if (*o*, *r*) is an arc in *N*. Note that, by Hall’s marriage theorem (Hall 1987), *B* has a matching that covers *O* if and only if each subset $S\subseteq O'$ has at least |*S*| neighbors in *B*.

To prove the theorem, assume first that *N* is strongly tree-based. Consider any rooting *D* of *N* with the same vertex set as *N* (which exists because any rooting where the root subdivides an edge or arc of *N* can be easily modified to obtain a rooting in which the root is a vertex of *N*). Let $\rho$ denote the sole root of *D*. We create a rooting $D'$ of *N* from *D* as follows. If $\rho$ is an omnian in *N* and $d_N^e(\rho )=1$ then subdivide the edge incident to $\rho$ in *N* by a new vertex $\rho '$, make $\rho '$ the root of $D'$, orient the edge between $\rho$ and $\rho '$ as $(\rho ',\rho )$, and retain the directions of the remaining arcs of *D*. Otherwise, define $D'$ to be *D*. By construction of $D'$, for each omnian $o\in O$ of *N*, we have that all outgoing arcs of *o* in *D* are also outgoing arcs of *o* in *N*. Since *N* is strongly tree-based, $D'$ is tree-based. Consider a base tree $T'$ of $D'$. Then, $T'$ contains at least one outgoing arc $a_o$ (in $D'$), for each $o\in O$ (the omnians of *N*). In addition, $T'$ contains exactly one incoming arc of each reticulation in *R*. Hence, the arcs $a_o$, $o\in O$, form a matching in *B* that covers *O*. By Hall’s marriage theorem recalled above, this implies that each $S\subseteq O$ has at least |*S*| neighbors in *B* and hence that $|\delta ^+(S)|\ge |S|$.

Conversely, assume that for each $S\subseteq O$ we have that $|\delta ^+(S)|\ge |S|$. Then, by Hall’s marriage theorem, *B* has a matching *M* that covers *O*. Consider any rooting *D* of *N*. Let $\rho$ denote the sole root of *D*. We construct a rooted spanning tree *T* of *D* with leaf set *L*(*D*) as follows, see Fig. 8. For each $o\in O$ consider the reticulation $r\in R$ that *o* is matched to by *M*. Note that (*o*, *r*) may not be an arc of *D* because it could have been subdivided by the root. If (*o*, *r*) is an arc of *D*, then include it in *T*. Otherwise, *D* contains an arc $(\rho ,r)$ which we include in *T*. For each reticulation in *R* that does not have an incoming arc in *T* yet, choose one incoming arc arbitrarily and add it to *T*. Finally, add all arcs whose heads are not reticulations

also to *T*. Clearly, *T* is a rooted spanning tree of *D*.

It remains to show that *T* has leaf set *L*(*D*). Clearly, each leaf of *D* is a leaf of *T*. Suppose *T* has a leaf *v* that is not a leaf in *D*. Then, in *D*, *v* has at least one outgoing arc and the head of every outgoing arc of *v* is a reticulation.

Hence, in *N*, *v* has exactly one incident edge, no incoming arcs and at least one outgoing arc. Thus, *v* is an omnian of *N*. This leads to a contradiction since *T* contains, for each omnian of *N*, at least one outgoing arc of *D*. $\square$

Intriguingly, characterizing semi-directed networks that are weakly tree-based requires a different approach (see Corollary 4 at the end of Sect. 7).

## Cherry picking

In this section we consider another concept, called “cherry picking”, that can be used to characterize certain multi-semi-directed networks. More specifically, we focus on how cherry picking can be applied to characterize weakly and strongly orchard multi-semi-directed networks, classes of networks that can be used to model lateral gene transfer (see e.g. van Iersel et al. 2022). To state the main results (Theorems 7 and 8), we require some further definitions. Recall that a *cherry* of a mixed graph is an ordered pair of leaves (*x*, *y*) such that there is a length-2 path between *x* and *y*, either consisting of two edges or of two arcs directed towards *x* and *y*, respectively. *Reducing* a cherry (*x*, *y*) is defined as deleting *x* and suppressing any resulting non-root degree-2 vertex.

To introduce cherry picking for multi-rooted networks, consider a multi-rooted network *D* on *X*. A *reticulated cherry*^4^ of *D* is an ordered pair of leaves (*x*, *y*) such that *y* is a reticulation leaf and there is a length-3 path between *x* and *y*. *Reducing* a reticulated cherry (*x*, *y*) of *D* means deleting the arc from the parent of *x* to the parent of *y* and suppressing any resulting non-root degree-2 vertices. Let *D*(*x*, *y*) be the result of reducing a cherry or reticulate cherry (*x*, *y*) in *D*. Then the leaf set of *D*(*x*, *y*) is either the same as the leaf set of *D*, or, if (*x*, *y*) is a cherry of *D*, then the leaf set of *D*(*x*, *y*) is $X\setminus \{x\}$. We say that *D* is *orchard* if it can be reduced to a disjoint union of arcs using a sequence of cherry reductions and reticulated cherry reductions.

**Fig. 9:**
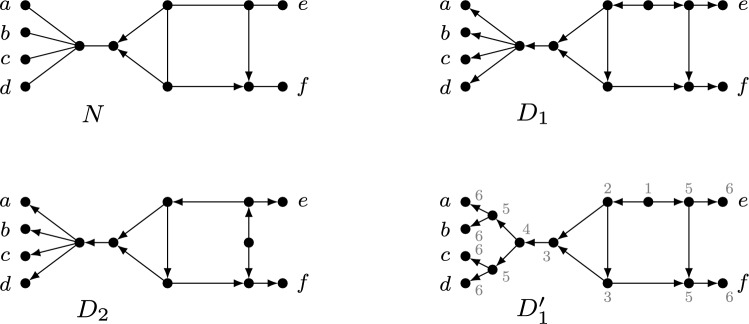
A semi-directed network *N* on $X=\{a,b,c,d,e,f\}$ that is weakly orchard but not strongly orchard, along with a rooting $D_1$ that is orchard and a rooting $D_2$ that is not orchard. The rooted network $D'_1$ is a binary resolution of $D_1$ with the gray numbers indicating an HGT-consistent labelling for $D'_1$

Furthermore, we say that a multi-semi-directed network *N* is *weakly orchard* if *N* has a rooting that is orchard and that *N* is *strongly orchard* if every rooting of *N* is orchard. See Fig. 9 for an example of a semi-directed network *N* that is weakly orchard but not strongly orchard.

**Fig. 10:**
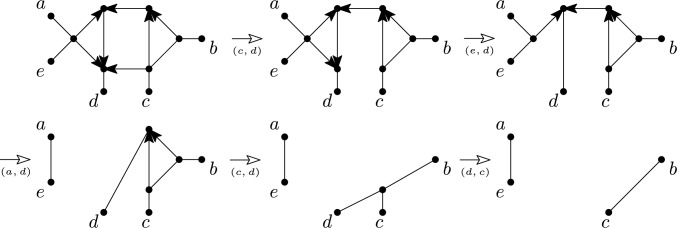
Example of a weakly orchard multi-semi-directed network on ${X=}\{a\ldots , e\}$ and a sequence of cherry reductions and reticulated cherry reductions. In each case, the (reticulated) cherry that is reduced is indicated below the arrow that indicates the reduction

**Fig. 11:**
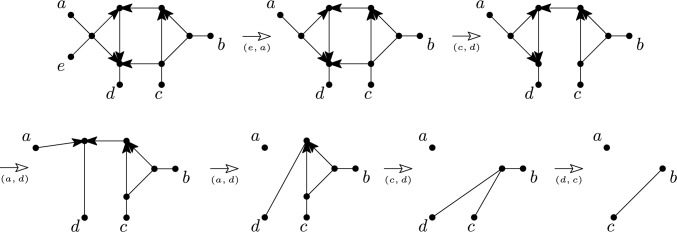
An alternative sequence of cherry reductions and reticulated cherry reductions for the weakly orchard multi-semi-directed network on $X=\{a\ldots , e\}$ from Fig. 10. In each case, the (reticulated) cherry that is reduced is indicated below the arrow that indicates the reduction. Note that in the bottom-left multi-semi-directed network, *a* has become a root instead of a leaf and that (*a*, *d*) is a reticulated cherry in that network since $a \in X.$

We now define reticulated cherries and their reductions for a multi-semi-directed network *N* on *X*. This definition is slightly different from the definition above for multi-rooted networks since *N* may have elements of *X* that are roots, see for example root *e* in the 2-semi-directed network in Fig. 3. A *reticulated cherry* of *N* is an ordered pair (*x*, *y*) with $x,y\in X$ such that *y* is a reticulation leaf and there is a length-2 or length-3 path between *x* and *y*. *Reducing* a reticulated cherry (*x*, *y*) of *N* is defined as deleting the arc between the neighbours of *x* and *y* or deleting the arc between *x* and the neighbour of *y* and suppressing any resulting non-root degree-2 vertices. See Figs. 10 and 11 for examples. For (*x*, *y*) a reticulated cherry or a cherry of *N*, let *N*(*x*, *y*) denote the resulting mixed graph that is the result of reducing (*x*, *y*) in *N*. Note that each connected component of *N*(*x*, *y*) is either an isolated vertex in *X* or a multi-semi-directed network on a subset of *X*.

Intriguingly, binary rooted networks that are orchard can be characterized in terms of a so-called HGT-consistent labelling (van Iersel et al. 2022). As it turns out, this concept can be canonically extended to binary multi-rooted networks. In turn, this provides us with a tool to characterize multi-semi-directed networks that are weakly orchard. To make this more precise, suppose that $D=(V,A,{\emptyset })$ is a binary multi-rooted network. Then we call a map $t:V\rightarrow \mathbb {N}$ a *HGT-consistent labelling* for *D* if

1. $t(u)\le t(v)$ for each arc $(u,v)\in A$;
2. $t(u) < t(v)$ for each arc $(u,v)\in A$ with *v* not a reticulation; and
3. for each reticulation *v* it holds that $t(u) = t(v)$ for exactly one parent *u* of *v*.

See Fig. 9 for an example of a HGT-consistent labelling of a 1-rooted network.

We call a rooting *D* of a multi-semi-directed network *N* on *X* *nice* if every root of *D* is either a vertex of *N* that is not in *X* or subdivides an arc of *N* whose tail is in *X*. We will use the following useful fact concerning nice rootings.

### Observation 1

Every multi-semi-directed network has a nice rooting.

### Proof

Suppose that *N* is a multi-semi-directed network. Consider an arbitrary rooting *D* of *N*. If a root $\rho$ of *D* is not a vertex of *N*, then it subdivides an edge *e* or an arc *a* of *N*. If $\rho$ subdivides *e*, we can make any vertex incident with *e* a root instead of $\rho$. If $\rho$ subdivides an arc *a* whose tail is not in *X*, then we can make the tail of *a* a root instead of $\rho$. $\square$

We now define a *binary resolution* of a multi-rooted network $N={(V,A)}$ which we shall need to state our next result. This is the binary multi-rooted network obtained from *N* by (i) replacing every vertex *v* with $d_N^+(v)\ge 3$ and its set of outgoing arcs by a rooted binary tree *T* with root *v*, so that all arcs in *T* are directed away from *v*, and the leaf set of *T* consists of those $w\in V$ such that $(v,w)\in A$, and (ii) replacing every vertex *w* in *N* with $d_N^-(w)\ge 3$ and its set of incoming arcs by a rooted binary tree *T* with root *w*, in which the directions of all arcs in *T* are reversed so that they are all directed towards *w* and the leaf set of *T* consists of those $v\in V$ with $(v,w)\in A$. See Fig. 9 for an example.

To simplify the exposition of the remainder of this section, we shall from now on assume, without loss of generality, that multi-rooted and multi-semi-directed networks have no isolated vertices.

### Theorem 7

Given a multi-semi-directed network *N* on *X*, the following are equivalent.

1. *N* is weakly orchard;
2. there exists a sequence of cherry reductions and reticulated cherry reductions that reduces *N* to a forest in which each tree is either a single edge whose two adjacent vertices are in *X* or a single vertex that is in *X*;
3. *N* has a rooting that has a binary resolution that admits an HGT-consistent labelling;
4. every nice rooting of *N* is orchard;
5. *N*(*x*, *y*) is weakly orchard for some cherry or reticulated cherry (*x*, *y*).

### Proof

That (4) implies (1) is trivial, given that *N* has at least one nice rooting by Observation 1.

We now show that (1) implies (3). If *N* is weakly orchard, then it has a rooting *D* that is orchard. Since *D* is a multi-rooted network, we can obtain a rooted network $D'$ from *D* by adding a new root $\rho$ with an arc to each root or *D*. Consider any sequence of cherry and reticulated cherry reductions that transforms *D* into a disjoint union *U* of arcs the heads of which are elements in *X*. The same sequence transforms $D'$ into a rooted star tree *S*, that is, the tree obtained from *U* as follows. First, add a root $\rho '$ to *U*. Next, add an arc from $\rho '$ to the tail $t_a$ of each arc *a* in *U*. Finally, suppress all vertices $t_a$. Clearly, *S* can easily be transformed into a single arc whose head is in *X* by cherry reductions. Hence, $D'$ is also orchard and, by [(van Iersel et al. 2022), Theorem 2], $D'$ has a binary resolution $D'_b$ that admits a HGT-consistent labelling. Deleting all vertices and arcs of $D'_b$ that where added to $D'$ to obtain a binary resolution of $\rho$ as part of obtaining $D_b'$ then gives a binary resolution of *D* with a HGT-consistent labelling, completing the proof that (1) implies (3). The converse direction, i.e. that (3) implies (1), can be shown in a similar manner.

We now prove that (1) implies (2). Let *D* be a rooting of *N* that is orchard. Consider a sequence $\sigma$ of cherry and reticulated cherry reductions that transforms *D* into a disjoint union of arcs the heads of which are elements in *X*. A cherry (*x*, *y*) that can be reduced in *D* can also be reduced in *N* unless the path between *x* and *y* in *D* contains a root of *D* and this root is not a vertex of *N* (i.e. it is suppressed when semi-deorienting *D* to obtain *N*). In this case, *N* contains a connected component consisting of only the edge $\{x,y\}$. A reticulated cherry (*x*, *y*) that can be reduced in *D* can also be reduced in *N*. The outcome of reducing (*x*, *y*) in *N* and in *D* in this case, respectively, is the same (up to taking the semi-deorientation) unless the path between *x* and *y* in *D* contains a root of *D* and this root is not a vertex of *N* (i.e. it is suppressed when semi-deorienting *D* to obtain *N*). In this case, the reduced version of *D* contains a connected component consisting of a single arc whose head is in *X*, while the reduced version of *N* contains a connected component consisting of a single vertex in *X*. Hence, $\sigma$ reduces *N* to a forest in which each connected component is either a single edge whose two incident vertices are in *X* or a single vertex that is in *X*.

We now show that (2) implies (4). Let *D* be a nice rooting of *N*. We prove by induction on the number *m* of vertices of *D* that *D* is orchard. Since $|X|\ge 2$, the bases case is $m=2$ and is trivial. Assume that the stated implication holds for all multi-semi-directed networks that have a nice rooting with $2\le l\le m$ vertices and that *N* is such that *D* has $m+1$ vertices. Let (*x*, *y*) be a cherry or reticulated cherry in *N*. Then (*x*, *y*) is also a cherry or reticulated cherry of *D* because *D* is a nice rooting of *N*. Note that it is possible that *N* contains a length-2 path between *x* and *y* while *D* contains a length-3 path between *x* and *y*. This can happen if $d(x)=d^+(x)=1$. In this case, $d_{N(x,y)}(x)=0$ and so *x* is an isolated vertex in *N*(*x*, *y*) while *D*(*x*, *y*) contains a connected component in the form of an arc from a root of *D*(*x*, *y*) to *x*. In this case, let $D',N'$ be *D*(*x*, *y*), *N*(*x*, *y*), respectively, with the connected component containing *x* removed. Otherwise, simply let $D'=D(x,y)$ and $N'=N(x,y)$. Then $D'$ is a nice rooting of $N'$. By induction, it follows that $D'$ is orchard, from which we can conclude that *D* is orchard.

By the equivalence of (1) and (2) shown above, it follows easily that (5) implies (1).

It remains to prove that (1) implies (5). Suppose that *N* is weakly orchard and that (*x*, *y*) is a cherry or reticulated cherry of *N*. By Observation 1, *N* has a nice rooting *D*. Then *D* is orchard by the equivalence of (1) and (4). Furthermore, (*x*, *y*) is a cherry or reticulated cherry also in *D*. Let $D'$ be the rooted network obtained from *D* by adding a new root $\rho$ with an arc to each previous root and suppressing any resulting non-root degree-2 vertices. Then (*x*, *y*) is also a cherry or reticulated cherry in $D'$ and $D'$ is also orchard. Hence, by [(Janssen and Murakami 2021), Proposition 1], $D'(x,y)$ is orchard. Thus, *D*(*x*, *y*) is orchard. It follows that *N*(*x*, *y*) has a rooting that is orchard. Hence, *N*(*x*, *y*) is weakly orchard. $\square$

From Theorem 7, it follows that one can decide in linear time whether a given multi-semi-directed network is weakly orchard since, just as for rooted networks, cherries and reticulated cherries can be reduced in arbitrary order.

To state the second main result of this section which concerns semi-directed networks, we require some further definitions. Suppose *N* is a semi-directed network on *X*. A *cherry picking sequence* of *N* is a sequence $({s_1},\ldots ,s_k)$ of ordered pairs of elements of *X*, such that ${s_1}$ is a cherry or reticulated cherry of $N_0:=N$ and, for all $i\in \{1,\ldots ,{k-1}\}$, the pair $s_{i+1}$ is a cherry or reticulated cherry in $N_i:=N_{i-1}(s_i)$ and $N_k:=N_{k-1}(s_k)$ is a graph in which each connected component is either an isolated vertex in *X* or an edge such that both incident vertices are contained in *X*. Note that in case we want to emphasize the order in which the pairs $s_i$ are reduced, we also write $N\circ (s_1,\ldots , s_i)$ for $N_i$.

### Lemma 4

Let *N* be a semi-directed network and let (*x*, *y*) be a cherry or a reticulated cherry of *N*. If *N* is strongly orchard then *N*(*x*, *y*) is strongly orchard.

### Proof

Let *D* be any rooting of *N*(*x*, *y*). Then a rooting $D'$ of *N* can be obtained from *D* by the following small modifications. If (*x*, *y*) is a cherry and *x* was deleted in the construction of *N*(*x*, *y*) from *N*, then $D'$ is obtained from *D* by subdividing the arc incident to *y* by a vertex *w* and adding leaf *x* with an arc (*w*, *x*). If (*x*, *y*) is a reticulated cherry, then $D'$ is obtained from *D* by subdividing the arc incident to *y* by a vertex *v* and the arc incident to *x* by a vertex *u* and adding an arc (*u*, *v*). Since *N* is strongly orchard, $D'$ is orchard. It then follows that *D* is orchard. Hence, *N*(*x*, *y*) is strongly orchard. $\square$

**Fig. 12:**
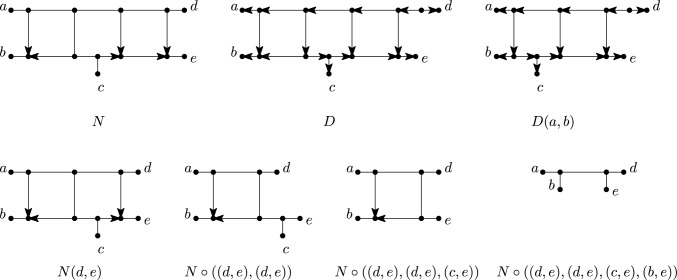
A semi-directed network *N* on $\{a,b,c,d,e\}$ that is weakly orchard but not strongly orchard, along with a rooting *D* that is not orchard. This is easy to see since *D* has no cherry, (*a*, *b*) is the only reticulated cherry of *D*, and the depicted network *D*(*a*, *b*) has no cherries or reticulated cherries. The bottom row of networks shows the first 4 networks obtain when reducing *N* by the strong cherry picking sequence ((*d*, *e*), (*d*, *e*), (*c*, *e*), (*b*, *e*), (*a*, *b*), (*b*, *d*)) of *N*

The converse of the Lemma 4 does not hold in general, see Fig. 12. Motivated by this, we define an *scr-cherry* (source component reticulated cherry) of a semi-directed network *N* as a reticulated cherry (*x*, *y*) such that *x* is in a source component of the network.

The next lemma, combined with Lemma 4, shows that cherries and reticulated cherries that are not scr-cherries can be reduced in arbitrary order.

### Lemma 5

Let *N* be a semi-directed network and let (*x*, *y*) be a cherry or a reticulated cherry of *N* that is not a scr-cherry. If *N*(*x*, *y*) is strongly orchard then *N* is strongly orchard.

### Proof

Assume that *N*(*x*, *y*) is strongly orchard. Let *D* be any rooting of *N*. We distinguish between the cases that (*x*, *y*) is a cherry or reticulated cherry in *D* or that this is not the case.

First suppose that (*x*, *y*) is a cherry or reticulated cherry in *D*. Then *D*(*x*, *y*) is a rooting of *N*(*x*, *y*) since (*x*, *y*) is not an scr-cherry. Since *N*(*x*, *y*) is strongly orchard, *D*(*x*, *y*) must be orchard. Hence, *D* is orchard. So *N* is strongly orchard.

Now suppose that (*x*, *y*) is neither a cherry nor a reticulated cherry of *D*. This can only happen if (*x*, *y*) is a cherry of *N* that is in a source component of *N* and the single root of *D* subdivides an edge on the path between *x* and *y* in *N*. Then modify *D* to a rooting $D'$ by making the internal vertex of this path the root. Then we can use the argument from the previous paragraph to show that *N* is strongly orchard. $\square$

### Remark

It follows from Theorem 7, Lemmas 4 and 5, and Fig. 12 that there is a key distinction between the weakly and strongly orchard properties concerning the reduction of (reticulated) cherries in arbitrary order. Specifically, the property of a multi-semi-directed network being weakly orchard is preserved under arbitrarily reducing both cherries and reticulated cherries. In contrast, a semi-directed network being strongly orchard is preserved only under arbitrarily reducing cherries and reticulated cherries that are not scr-cherries.

We call a cherry picking sequence $s=(s_1,\ldots ,s_k)$ of $N_0=N$ *strong* if, for each $i\in \{1,\ldots ,k\}$, it holds that if $N_{i-1}$ has at least one scr-cherry, then $s_i$ is an scr-cherry of $N_{i-1}$. Note that if $N_{i-1}$ has no scr-cherries then, by definition of a cherry picking sequence, $s_i$ is a cherry or reticulated cherry of $N_{i-1}$. We now state the second main result of this section.

### Theorem 8

Let *N* be a semi-directed network *N* on *X*. Then *N* is strongly orchard if and only if, for each strong cherry picking sequence $s=(s_1,\ldots ,s_k)$ of *N* and for each $i\in \{1,\ldots ,k\}$, it holds that if $s_i$ is an scr-cherry of $N_{i-1}=N\circ (s_1,\ldots ,s_{i-1})$ then $N_{i-1}$ has at least two scr-cherries where we put $N_0=N$.

### Proof

First suppose that *N* is strongly orchard and assume for contradiction that there exists a strong cherry picking sequence $s=(s_1,\ldots ,s_k)$ of *N* such that there exists some $1\le i\le k$ such that $s_i$ is a scr-cherry of $N_{i-1}=N\circ (s_1,\ldots ,s_{i-1})$ and $N_{i-1}$ has no other scr-cherries. Since *s* is strong, $N_{i-1}$ has no cherries or reticulated cherries apart from $s_i$. Since $s_i=(x,y)$ is an scr-cherry, there exists a rooting $D_{i-1}$ of $N_{i-1}$ where the root subdivides the edge of $N_{i-1}$ incident to *x*. Consequently, $D_{i-1}$ has no cherries or reticulated cherries and is therefore not orchard. Hence, $N_{i-1}$ is not strongly orchard and, by Lemma 4, *N* is not strongly orchard, a contradiction.

The other direction of the proof is by induction on $k=|X|+|R|-2$, with *R* the set of reticulations of *N*. For $k=0$ the statement is trivially true. Assume $k\ge 1$ and that for each strong cherry picking sequence $s=(s_1,\ldots ,s_k)$ of $N_0=N$ and for each $i\in \{1,\ldots ,k\}$, it holds that if $s_i$ is an scr-cherry of $N_{i-1}=N\circ (s_1,\ldots ,s_{i-1})$ then $N_{i-1}$ has at least two scr-cherries.

First suppose that *N* has a cherry or a reticulated cherry (*x*, *y*) that is not an scr-cherry. Since *N*(*x*, *y*) is strongly orchard by induction, it follows that *N* is strongly orchard by Lemma 5.

Now suppose that *N* has no cherries or reticulated cherries that are not scr-cherries. Then *N* has at least two scr-cherries (*x*, *y*) and (*w*, *z*) (possibly, $z=y$). Consider any rooting *D* of *N*. Then at least one of (*x*, *y*) and (*w*, *z*) is a reticulated cherry in *D*. Assume without loss of generality that (*x*, *y*) is a reticulated cherry of *D*. Since *N*(*x*, *y*) is strongly orchard by induction, each rooting of *N*(*x*, *y*) is orchard. In particular, *D*(*x*, *y*) is orchard. Hence, *D* is orchard. Since *D* was arbitrary, it follows that *N* is strongly orchard. $\square$

The example in Fig. 12 shows why in the characterization in Theorem 8 “for each” cannot be replaced by “there exists”. Even though there exists a cherry picking sequence of the required type in the depicted semi-directed network *N*, *N* is not strongly orchard. Indeed, *N* also has a strong cherry picking sequence that is not of the required type.

## Path partitions

We now turn to our final concept for characterizing classes of (multi-)semi-directed networks called path partitions, see also Francis et al. (2018); Lafond and Moulton (2025); Huber et al. (2022). Basically speaking, for a multi-rooted network, this is a partition of the vertex set of the network whose parts induce a collection of directed paths each of which must end in a leaf. Multi-rooted networks that enjoy this property are called *forest-based* networks and can be used to model introgression (Scholz et al. 2019); they are considered in more depth in Lafond and Moulton (2025); Huber et al. (2022). In this section, we extend the theory of path partitions from multi-rooted networks to (multi-)semi-directed networks and shall see that having a path partition characterizes weakly tree-based semi-directed networks (Corollary 4).

**Fig. 13:**
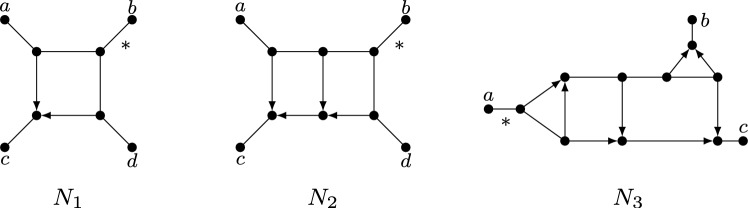
The semi-directed network $N_1$ on $X = \{a,b,c,d\}$ is weakly tree-child, weakly forest-based and weakly tree-based. In each case, the rooting is obtained by subdividing the edge labelled $*$ to obtain the root. The semi-directed network $N_2$ on *X* is weakly forest-based and weakly tree-based (since the rooting with the root subdividing the edge labelled $*$ is forest-based and tree-based), but not weakly tree-child. The semi-directed network $N_3$ on $\{ a,b,c\}$ is weakly tree-based (since the rooting with the root subdividing the edge labelled $*$ is tree-based), but not weakly tree-child and not weakly forest-based

We begin with some definitions. Suppose that *G* is a mixed graph. We call *G* a *forest* if it does not contain a cycle. If *D* is a multi-rooted network on *X*, then we call *D* *forest-based* if there exists a subgraph *F* of *D* in the form of a forest such that *F* spans *V*(*D*) and has leaf set *X* and, for each arc $(u,v)\in A(D)\setminus A(F)$, the vertices *u* and *v* are in different trees of *F*. In this case, we call *F* a *support forest* of *D*. Note that an element $T\in F$ might be a single vertex or *T* might contain a vertex *v* such that $d_T(v)=2$. If *N* is a multi-semi-directed network on *X*, then we say that *N* is *weakly forest-based* if *N* has a rooting *D* that is forest-based.

**Fig. 14:**
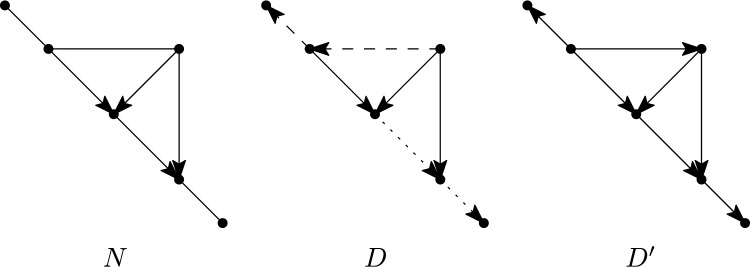
A multi-semi-directed network *N* that is weakly forest-based but not strongly forest-based, along with a rooting *D* of *N* that is forest-based (the dashed and dotted paths are the trees that make up the support forest) and a rooting $D'$ of *N* that is not forest-based

Note that since the leaf sets of *D* and *N* coincide, no leaf of *N* can be a root in *D*. If every rooting of *N* with leaf set *X* is forest-based, then we call *N* *strongly forest-based*. See Fig. 14 for some examples to illustrate these definitions. Note if *N* is semi-directed, then by [(Huber et al. 2022), Theorem 1] it follows that if *N* is weakly (resp. strongly) forest-based then it is weakly (resp. strongly) tree-based, but not conversely (see Fig. 13).

**Fig. 15:**
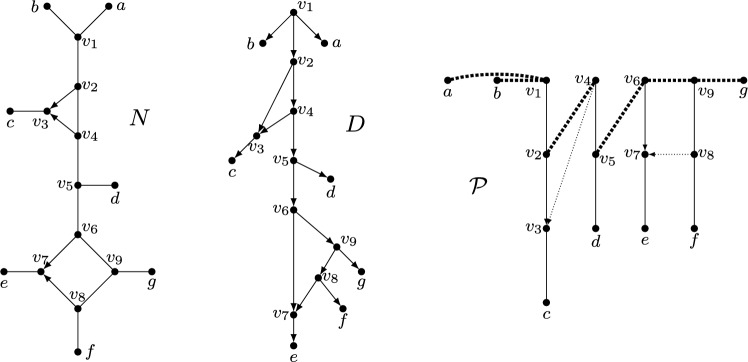
A semi-directed network *N* on $X = \{a, \ldots , g\}$ that is weakly forest-based as *D* is a rooting of *N* that is forest-based. The trivial paths (*a*), (*b*) and (*g*) along with the four semi-directed paths made up by the thin black edges and arcs form a collection of semi-directed paths $\mathcal {P}$ in *N* that satisfies Properties (P1)-(P3) of Lemma 6. The vertices *a*, *b*, $v_1$, $v_4$, $v_6$, $v_9$ and *g* are the handy vertices of the paths that contain them. The dotted thick black edges make up the three cross components of $\mathcal {P}$, while the thin black, dotted arcs are neither in a semi-directed path of $\mathcal {P}$ nor in a cross component

Now, suppose that *N* is a multi-semi-directed network on *X*. For $x \in X$, let *P* be either a semi-directed path $(u_1=u,u_2,\ldots , u_k=x)$, $k\ge 2$, in *N* joining a vertex $u\in V(N)-X$ to *x* or the trivial path $(u=x)$. Then we call *u* the *handy vertex* of *P*. In addition, for a collection $\mathcal {P}$ of semi-directed paths in *N*, we call a maximal connected subgraph containing only edges $\{u,v\}$ of *N* with *u*, *v* in different semi-directed paths of $\mathcal {P}$ a *cross component* of $\mathcal {P}$. We now show that a special type of path partition arises from weakly forest-based multi-semi-directed networks (see Fig. 15).

### Lemma 6

Let *N* be a multi-semi-directed network *N* on *X* that has a rooting *D* with leaf set *X* that is forest-based. Then there exists a collection $\mathcal {P}$ of semi-directed paths in *N* such that

1. each vertex of *N* is in exactly one semi-directed path in $\mathcal {P}$;
2. each semi-directed path in $\mathcal {P}$ is either a trivial path (*x*) with $x\in X$ or a semi-directed path from a vertex in $V(N)-X$ to some element in *X*;
3. each cross component of $\mathcal {P}$ contains at most one vertex that is not the handy vertex of a semi-directed path in $\mathcal {P}$.

Moreover, if *F* is a support forest for *D* then, for each arc $(u,v) \in A(D) \setminus A(F)$, we have that

(P4) if $\{u,v\}$ is an edge or (*u*, *v*) is an arc of *N* and $u,v\in P\in \mathcal {P}$ then *u* and *v* appear consecutive on *P*.

### Proof

Suppose that *F* is a support forest for *D*. We can decompose *F* into a set $\mathcal S$ of (directed) paths by, for each vertex of *F* with outdegree greater than 1 in *F*, arbitrarily deleting all but one outgoing arc from *F*. Let $\mathcal {P}$ denote the collection of semi-directed paths in *N* that corresponds to $\mathcal S$. Note that the first arc of a path in $\mathcal S$ might be different from the first arc of the corresponding semi-directed path in $\mathcal {P}$. We show that Properties (P1)-(P3) are satisfied by $\mathcal {P}$.

Clearly, $\mathcal {P}$ satisfies Properties (P1) and (P2). To see that $\mathcal {P}$ satisfies Property (P3), we claim first that for every edge *e* in a cross component of $\mathcal {P}$ one of the vertices incident with *e* must be the handy vertex of a semi-directed path in $\mathcal {P}$. To see this, assume for contradiction that *e* is an edge in a cross component of $\mathcal {P}$ and none of the vertices incident with *e*, call them *u* and *v*, is the handy vertex of a semi-directed path in $\mathcal {P}$. Then *u* and *v* must be an interior vertex of the semi-directed path in $\mathcal {P}$ that contains it, respectively. Since *F* is a forest spanning *V*(*D*), it follows that one of *u* and *v* must be a reticulation in *N*. Hence (*u*, *v*) or (*v*, *u*) must be an arc in *N*; a contradiction as $\{u,v\}$ is an edge in *N*. Hence, the claim holds

Assume for contradiction, that Property (P3) does not hold. Then there exists a cross component *C* of $\mathcal {P}$ that contains two or more vertices that are not the handy vertex of the semi-directed path in $\mathcal {P}$ that contains them. Let *u* and $u'$ denote two such vertices of *C* and let *v* and $v'$ be the vertices of *N* such that $\{u,v\}$ and $\{u', v'\}$ are edges in *C* and *u* and *v* are in different semi-directed paths of $\mathcal {P}$ and $u'$ and $v'$ are in different semi-directed paths of $\mathcal {P}$. Note that the semi-directed paths in $\mathcal {P}$ that contain *u* and $u'$ might be the same. Then, by the previous claim, *v* and $v'$ must be the handy vertices of semi-directed paths in $\mathcal {P}$. Since, by Theorem 2, *N* cannot contain a semi-directed cycle, it follows that *C* is a tree. Therefore, there exists a path *U* in *C* joining *u* and $u'$. Replacing, if necessary, *v* with the vertex on *U* adjacent with *u* and $v'$ with the vertex on *U* adjacent with $u'$, it follows that *U* has the form $(u, v, \ldots , v', u')$.

Since both $\{u,v\}$ and $\{u', v'\}$ are edges of *N* and so must be oriented in any rooting of *N* and, in combination, Properties (P1) and (P2) imply that any arc on a path *P* in $\mathcal {P}$ is oriented towards the handy vertex of *P*, it follows that $\{u,v\}$ and $\{u', v'\}$ must be oriented towards each other in the orientation of *C* induced by *D*. If $v\not =v'$ this is not possible since it implies that some edges in *U* must have been arcs in *N*. If $v=v'$ then *v* is a reticulation in *N*. Hence, *N* contains the arcs (*u*, *v*) and $(u',v)$ as it is a multi-semi-directed network; a contradiction since, by assumption, $\{u,v\}$ and $\{u',v\}$ are edges in *C* and therefore in *N*.

The remainder is an immediate consequence of the fact that *F* is a support forest for *D*. $\square$

Using Lemma 6, we can now characterize multi-semi-directed networks that are weakly forest-based (note that this is an analogue of [(Huber et al. 2022), Theorem 1]).

### Theorem 9

A multi-semi-directed network *N* on *X* is weakly forest-based if and only if there exists a collection $\mathcal {P}$ of semi-directed paths in *N* satisfying Properties (P1)-(P4) of Lemma 6.

### Proof

By Lemma 6, it suffices to show that if *N* has a collection of semi-directed paths that satisfies Properties (P1)-(P4) then *N* must be weakly forest-based. So suppose that $\mathcal {P}$ is a collection of semi-directed paths in *N* that satisfies these properties.

We start with associating a directed graph *D* to *N* and then show that *D* is in fact a rooting of *N* that is forest-based.

To obtain *D*, we employ Property (P2) and orient, for each non-trivial semi-directed path $P\in \mathcal {P}$, all edges on *P* towards the unique element in *X* it contains.

For each cross component *C* in $\mathcal {P}$, we do the following. Let $v_C$ be the unique vertex in *C* that is not a handy vertex of a semi-directed path in $\mathcal {P}$ (Property (P3)), if it exists. Otherwise, choose an arbitrary non-leaf vertex *v* in *C* to play the role of $v_C$.

In either case, we then orient all edges in *C* away from $v_C$. Observe that this is well-defined since *C* is a tree by Theorem 2. The obtained directed graph is *D*.

We now show that *D* is a rooting of *N*. To this end, we need to show that *D* is a multi-rooted network such that *N* is a semi-deorientation of *D*. Clearly, *D* is a multi-rooted network since it cannot contain a directed cycle as otherwise *N* would have contained a semi-directed cycle, which is not allowed by Theorem 2. To see that *N* is a semi-deorientation of *N*, we need to show that the reticulations of *D* are precisely the reticulations of *N*. Since every reticulation of *N* is also a reticulation of *D*, it suffices to show that every reticulation of *D* is also a reticulation of *N*. Assume for contradiction that *D* contains a reticulation *r* that is *not* a reticulation of *N*. Then since neither a leaf nor a root of *N* can be a reticulation in *D*, it follows that $d^e_N (r) = d_N (r) \ge 3$. Hence, at least one of the edges incident to *r* in *N* is in a cross component *C* of $\mathcal {P}$, and so *r* is a vertex in *C*. By Property (P3), it follows that *r* is a handy vertex of some semi-directed path in $\mathcal {P}$ or $r = v_C$ holds. Hence, *C* is oriented in *D* in such a way that *r* has exactly one incoming arc. Consequently, *r* cannot be a reticulation in *D*, a contradiction. Thus, *D* must be a rooting of *N*.

It remains to show that *D* is forest-based. Let $\mathcal {P}'$ denote the collections of semi-directed paths in *D* induced by $\mathcal {P}$. Then Property (P1) implies that $\mathcal {P}'$ is a forest spanning *V*(*D*). Furthermore, Property (P2) implies that the leaf set of *D* is *X*. Lastly, Property (P4) implies that, for each arc $a \in A(D) \setminus A(\mathcal {P}')$, the head of *a* and the tail of *a* are in different semi-directed paths of $\mathcal {P}'$. Hence, $\mathcal {P}'$ is a support forest for *D*. It follows that *N* is weakly forest-based. $\square$

As it turns out, in multi-semi-directed networks that are forest-based, collections of semi-directed paths that satisfy Properties (P1)-(P3) in Lemma 6 also turn out to hold the key for our characterization of semi-directed networks that are weakly tree-based.

### Corollary 4

A semi-directed network *N* on *X* is weakly tree-based if and only if there exists a collection $\mathcal {P}$ of semi-directed paths in *N* satisfying Properties (P1)-(P3) of Lemma 6.

### Proof

By the definition of weakly tree-based and Lemma 6, it suffices to show that if *N* contains a collection of semi-directed paths that satisfy Properties (P1)-(P3) of Lemma 6 then *N* is weakly tree-based. Suppose that $\mathcal {P}$ is a collection of semi-directed paths in *N* that satisfies these properties

We create a rooting *D* of *N* as in the proof of Theorem 9. Note that *D* now has a single root because *N* is semi-directed. To see that *D* is tree-based, observe that $\mathcal {P}$ again spans *V*(*D*) (by Property (P1)) and has leaf set *X* (by Property (P2)). By [(Francis et al. 2018), Theorem 2.1], this means that *D* is tree-based. Hence, *N* is weakly tree-based. $\square$

## Discussion

In this paper, we have introduced new explicit mathematical characterizations of multi-semi-directed networks, overcoming the need to implicitly define such networks through their rooted counterparts. By extending existing concepts for rooted networks—such as cherry picking sequences, omnians and path partitions—we have been able to explicitly characterize when a multi-semi-directed network has a rooting that is within some commonly studied classes of rooted networks. In particular, with the growing interest in semi-directed networks (see e.g. Baños 2019; Gross and Long 2018; Gross et al. 2021; Linz and Wicke 2023; Jingcheng and Ané 2023; Holtgrefe et al. 2025), our characterizations have the potential to make the mathematical analysis of the algebraic models associated with semi-directed networks more tractable (see e.g. Maxfield et al. 2025; Englander et al. 2025; Allman et al. 2025a).

Although we have presented some characterizations for when a multi-semi-directed network is contained within a certain class, there remain some open questions in this direction. For example, generalizing our characterization of strongly orchard semi-directed networks (Theorem 8) to multi-semi-directed networks, deciding whether or not strongly forest-based networks can be characterized with path partitions, and seeing if weakly orchard networks can be characterized in terms of an HGT-consistent labelling directly applied to the multi-semi-directed network are all interesting questions. In addition, similar questions could be investigated for other well-known network classes, including but not limited to *proper-forest-based*, *normal*, *reticulation visible* and *tree-sibling* networks (Kong et al. 2022).

Finally, our results open up a number of interesting algorithmic questions. We have already sketched an efficient algorithm to check if a mixed graph is a (multi-)semi-directed network (see the end of Sect. 4). Developing efficient algorithms to check whether a given (multi-)semi-directed network lies within a fixed class would be a logical next step. For some classes efficient algorithms follow directly from our results (see e.g. Theorem 5). However, for other classes the existence of efficient algorithms is not immediately obvious. For example, it would be interesting to determine whether or not there exists a linear time algorithm to check if a semi-directed network is strongly orchard. This question can be answered affirmatively for rooted networks, but for semi-directed networks a naive algorithm takes quadratic time (by checking every possible rooting).

## Data Availability

No datasets were generated or analysed during the current study.

## Footnotes

1 Rootings were called “rooted partners” in Maxfield et al. (2025); Linz and Wicke (2023).

2 From a biological perspective, turning a leaf in a (multi-)semi-directed network into a root of a (multi)-rooted network may seem problematic. However, since this only applies to leaves of a (multi-)semi-directed network not in *X*, which do not correspond to taxa, this does not pose a problem. A leaf of a (multi-)semi-directed network contained in *X* cannot be turned into a root of a (multi)-rooted network. Instead, its incident edge can be subdivided by a new vertex that becomes a root.

3 Source components were called “root components” in Maxfield et al. (2025).

4 Note that this definition is only for multi-rooted networks. Reticulated cherries in multi-semi-directed networks are defined in a slightly different way below.
